# Time series analysis of mumps and meteorological factors in Beijing, China

**DOI:** 10.1186/s12879-019-4011-6

**Published:** 2019-05-17

**Authors:** Yu Hao, Ran-ran Wang, Ling Han, Hong Wang, Xuan Zhang, Qiao-ling Tang, Long Yan, Juan He

**Affiliations:** 1 School of Traditional Chinese Medicine, Beijing University of Chinese Medicine, No. 11, Bei San Huan East Road, Chaoyang District, Beijing, 100029 China; 2 Hong Kong Chinese Medicine Clinical Study Centre, Chinese Clinical Trial Registry (Hong Kong Center), School of Chinese Medicine, Hong Kong Baptist University, Hong Kong, China

**Keywords:** Beijing, Meteorological factors, Mumps, Time series

## Abstract

**Background:**

Over the past decades there have been outbreaks of mumps in many countries, even in populations that were vaccinated. Some studies suggest that the incidence of mumps is related to meteorological changes, but the results of these studies vary in different regions. To date there is no reported study on correlations between mumps incidence and meteorological parameters in Beijing, China.

**Methods:**

A time series analysis incorporating selected weather factors and the number of mumps cases from 1990 to 2012 in Beijing was performed. First, correlations between meteorological variables and the number of mumps cases were assessed. A seasonal autoregressive integrated moving average model with explanatory variables (SARIMAX) was then constructed to predict mumps cases.

**Results:**

Mean temperature, rainfall, relative humidity, vapor pressure, and wind speed were significantly associated with mumps incidence. After constructing the SARIMAX model, mean temperature at lag 0 (β = 0.016, *p* < 0.05, 95% confidence interval 0.001 to 0.032) was positively associated with mumps incidence, while vapor pressure at lag 2 (β = ˗0.018, *p* < 0.05, 95% confidence interval ˗0.038 to ˗0.002) was negatively associated. SARIMAX (1, 1, 1) (0, 1, 1)_12_ with temperature at lag 0 was the best predictive construct.

**Conclusions:**

The incidence of mumps in Beijing from 1990 to 2012 was significantly correlated with meteorological variables. Combining meteorological variables, a predictive SARIMAX model that could be used to preemptively estimate the incidence of mumps in Beijing was established.

**Electronic supplementary material:**

The online version of this article (10.1186/s12879-019-4011-6) contains supplementary material, which is available to authorized users.

## Background

Mumps is a viral respiratory disease that is most likely to occur in children and adolescents. Parotid non-suppurative inflammation and painful swelling of the parotid gland are the main clinical features of mumps. The complications of mumps include meningoencephalitis, meningitis, orchitis, pancreatitis, and ovarian inflammation [1].

The mumps virus is a single-stranded RNA virus of the paramyxovirus family. It is a moderately to highly contagious virus that only infects humans. The main routes of transmission include direct contact, droplet propagation, and contact with contaminated objects [2]. Administration of the well-established live attenuated vaccine is the primary measure used to prevent mumps.

As at November 2016, of the 192 World Health Organization member states 127 (57%) included mumps vaccine in their national vaccination schedules [3]. Mumps-containing vaccines were introduced in China in 1990. Since 2008 the measles, mumps, and rubella (MMR) vaccine has been included in China’s national immunization programs [4]. There have been outbreaks of mumps in many countries including the United States [5], Canada [6], Czech Republic [7], Belgium [8], Portugal [9], and Serbia [10], even in populations that were vaccinated [11]. A total of 698,092 cases of mumps were reported in mainland China from 2013 to 2015, with an average annual incidence of 17.2 per 100,000 [12].

Climate change plays an important role in the spread of many infectious diseases, especially vector-borne and water-borne infectious diseases [13]. As early as 2500 years ago, Hippocrates observed the influences of climate change on gastrointestinal infections, tuberculosis, and central nervous system infections [14]. In some recent studies the onset of some infectious diseases including mumps was associated with specific changes in meteorological factors. In a study conducted in Guangzhou in China the incidence of mumps was positively correlated with mean temperature, relative humidity, and wind velocity, and negatively correlated with atmospheric pressure [15]. In a study in Taiwan, which is at a similar latitude to Guangzhou, the occurrence of mumps was significantly correlated with increased temperature and vapor pressure [16]. In a study in Fukuoka Prefecture in Japan the number of pediatric mumps cases increased significantly with increased average temperature and relative humidity [17]. In Jining in China, mumps occurrence was reportedly positively associated with temperature, wind speed, and sunshine duration, and negatively associated with relative humidity [18]. However, large-scale weather changes collectively incorporated into the so called North Atlantic Oscillation phenomenon were reportedly not crucial factors in fluctuations in annual mumps incidence rates in the Czech Republic [19]. The studies described above indicate similarities in different regions that may be related to the locations and climates of the study areas. To date there is no reported study on correlations between mumps incidence and meteorological parameters in Beijing, China.

The purpose of the present study was to assess correlations between weather factors and the incidence of mumps in Beijing, and to establish an accurate model for estimating epidemic trends pertaining to mumps.

## Methods

### Study area

Beijing is the capital of China. The city is located in the northern part of the vast North China Plain (39.9° N, 116.3° E), and it is situated in a zone of typical continental monsoonal climate with four clearly distinct seasons. Spring is windy, summer is hot and rainy, autumn is dry, and winter is cold [20].

### Data collection

#### Mumps data

Mumps has been a legally notifiable disease in China since September 1989. The monthly mumps data used in this study were obtained from the Chinese Center for Disease Control and Prevention. The number of mumps cases recorded from January 1990 to December 2012 was 197,726, and the diagnoses were based on the criteria used by the National Health and Family Planning Commission of the People’s Republic of China (formerly the Ministry of Health of the People’s Republic of China).

#### Meteorological data

Daily temperature, rainfall, relative humidity, vapor pressure, and wind speed data from 1989 to 2012 were provided by the Beijing Meteorological Bureau. Monthly means of the daily average values of these meteorological characteristics were calculated. The data used in this study are provided as Additional files 1.

### Statistical analysis

A descriptive analysis was conducted to assess the distribution of mumps cases and weather factors in Beijing. Seasonal autoregressive integrated moving average (SARIMA) models were then used to evaluate relationships between monthly numbers of mumps cases and meteorological parameters. SARIMA models were optimal for use in this study because seasonal and non-seasonal trends could be studied [21]. A SARIMA model is described as an autoregressive integrated moving average, p, d, and q, multiplied by P, D, and Q—where the non-seasonal parameters are the number of autoregressive terms (p), the number of differences (d), and the moving average (q), and the seasonal parameters are the number of seasonal autoregressive terms (P), the number of seasonal differences (D), and the seasonal moving average (Q).

The SARIMA model with explanatory variables (SARIMAX) extends the capability of the SARIMA model by integrating external information such as rainfall, wind speed, and other meteorological variables into a time series model [22]. In the present study a SARIMAX model was constructed to investigate mumps cases. The specific method used to generate the model is described below.

The first step was stabilization processing of the sequence. The data were processed by difference and conversion if the sequence was unstable or had a seasonal distribution. The second step was model identification. The order of p, P, q, and Q in the model was determined based on a graph of the autocorrelation function (ACF) and the partial autocorrelation function (PACF). For the pure autoregressive model (p), moving average model (q), and autoregressive moving average model (p, q), the parameter q could not exceed the lag of the ACF, and the parameter p did not exceed the lag of the PACF. The orders of d and D respectively represented the non-seasonal and seasonal difference times [23]. The model parameters were then estimated and verified. The maximum likelihood method and *t*-tests were used to estimate and test the model parameters. Akaike information criterion (AIC) values were used to measure the model fit. Smaller AIC values indicated a better model fit.

Model diagnostics were then performed. The Ljung-Box Q test was conducted to ascertain whether the residual series were random. A *p* value less than 0.05 suggested that the residual sequence was not white noise and that it contained information that was inadequately extracted. We then assessed the correlations between pairs of sequences with strong autocorrelations. Pre-whitened data were needed to separate the linear associations from their autocorrelations. Cross-correlation function (CCF) plots were used to evaluate relationships between the number of mumps cases and meteorological factors, and determine which covariates and lags were best for the model [24]. Only covariates that had significant parameter estimates and lowered the AIC value were selected [22]. Lastly, we incorporated the covariates into the model and repeated model parameter estimation/verification and model diagnostics to build the best SARIMAX model. We used the 1990–2010 data to construct the best SARIMA model and SARIMAX models. We then used the 2011–2012 data to assess the predictive capacity of the SARIMAX model. Data analysis was conducted using R 3.3.1 [21].

## Results

### Descriptive analysis

**Fig. 1:**
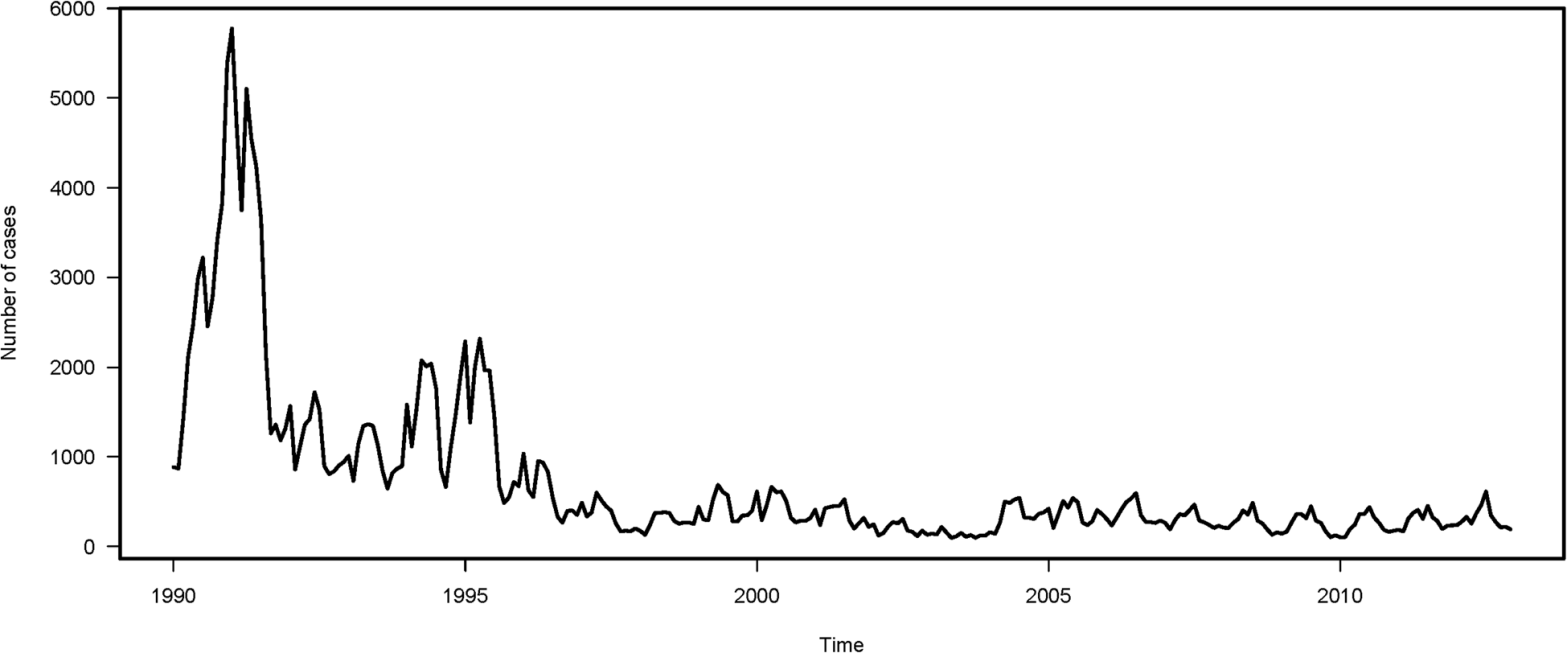
Time series plot of the numbers of mumps cases in Beijing, China, from 1990 to 2012

**Fig. 2:**
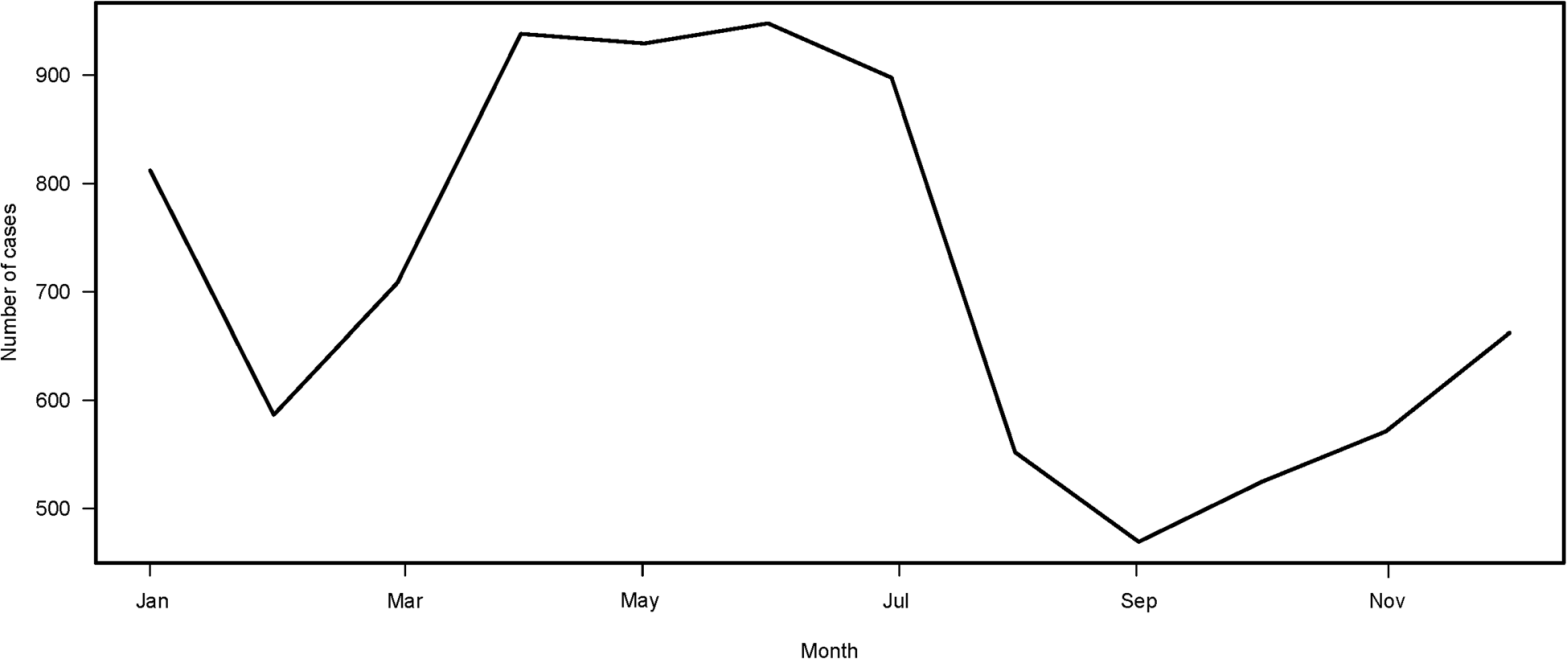
Monthly averages of mumps cases in Beijing, China, from 1990 to 2012

There were 197,726 reported mumps cases from 1990 to 2012 in Beijing. As shown in Fig. 1, the peak incidence occurred in 1992 with 38,979 cases, and the lowest incidence occurred in 2003 with 1579 cases. The seasonal variation is clearly depicted in Fig. 2. There was a major peak in the number of mumps cases in the late spring and early summer (April to July) and a minor peak in winter (December to January) during the years included in this study. More descriptive statistics pertaining to meteorological factors and numbers of mumps cases are shown in Table 1.

**Table 1:** Description of monthly average daily mean meteorological factors and monthly number of mumps case in Beijing, 1990-2012

Variables	Min	Max	Mean±SD	Median
Mean temperature(°C)	-6.43	31.44	13.14±10.70	14.39
Rainfall(mm)	0.00	14.81	1.46±2.06	0.62
Wind speed(m/s)	1.35	3.61	2.33±0.43	2.30
vapor pressure(hPa)	1.09	27.31	10.51±7.91	8.19
Relative humidity (%)	21.86	80.97	54.00±12.83	54.66
Number of mumps case	95	5775	716.40±923.13	362

### SARIMA model analysis

**Fig. 3:**
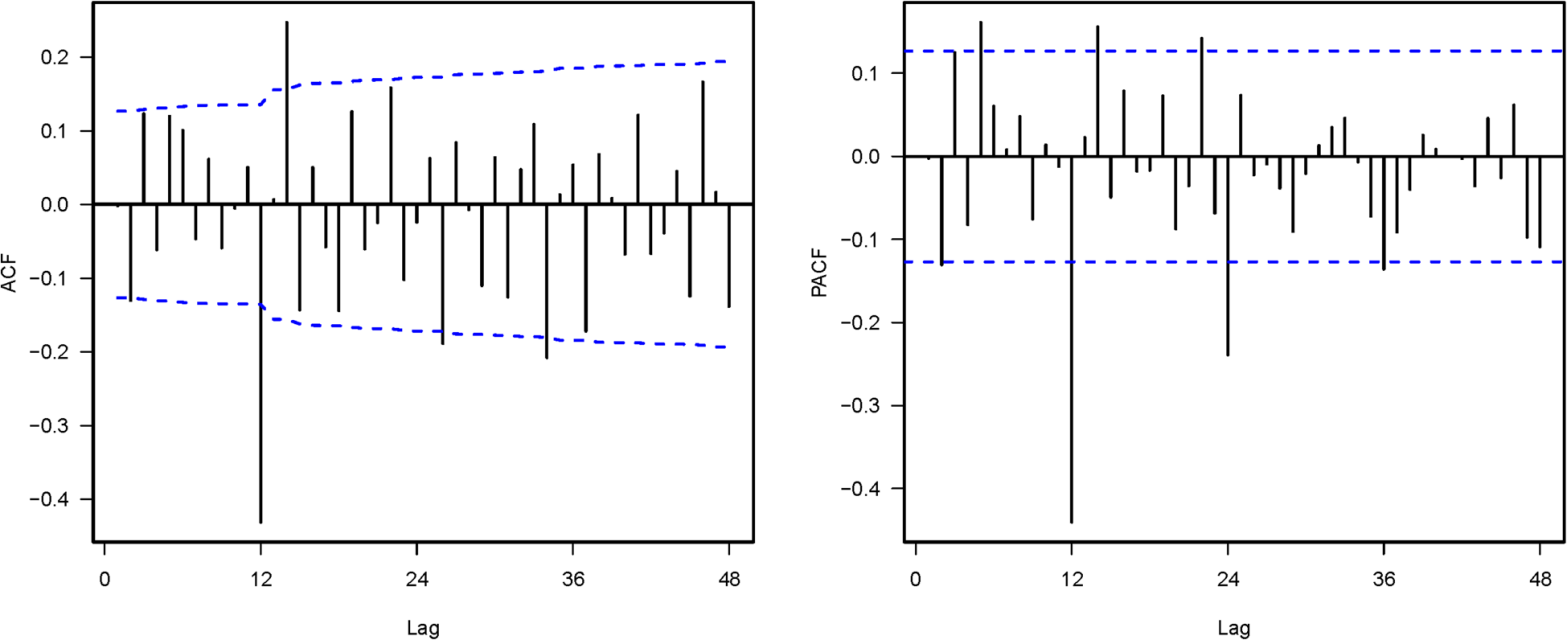
Results of autocorrelation function and partial autocorrelation function in time series analysis

A SARIMA model with 252 monthly data-points for mumps cases from 1990 to 2010 without any covariates was developed first. Mumps cases fluctuated within a large range over the time-course (Fig. 1). A logarithmic transformation of the time series of mumps cases was performed to stabilize fluctuations in the data. As the overall logarithmically transformed mumps data exhibited a downward trend and an obvious seasonal distribution, 1-step non-seasonal and 1-step seasonal differences were used separately. The value of both d and D was 1. The ACF and PACF of mumps cases are shown in Fig. 3. The ACF values of lag 2, 12, 14, 26, and 34 exceeded the critical value. The ACF value of lag 14, the neighbor of seasonal lag 12, was caused by the cross effect of the seasonal and non-seasonal autocorrelation. The significant ACF values of lag 26 and 34 may pertain to the presence of a year effect, though 26 months and 34 months are not strictly 2 years or 3 years. Therefore, the respective maximum values of the seasonal parameter Q and the non-seasonal parameter q were 1 and 2. Similarly, the sample PACF values were significant at lag 2, 5, 12, 14, 22, 24, and 36 (Fig. 3), so the respective maximum values of the seasonal parameter P and the non-seasonal parameter p were 3 and 5. We assumed that the maximum values of both P and p were 2 to make the model concise.

We searched all 54 SARIMA models that satisfied the conditions *p* ≤ 2, *P* ≤ 2, q ≤ 2, and Q ≤ 1 to find the most suitable model. Only two models yielded statistically significant parameters. Table 2 shows the results for these two models, Model A (SARIMA [1] [1, 1, 0]_12_) and Model B (SARIMA [1] [0, 1, 1]_12_). The AIC value of Model B (− 63.96) was lower than that of Model A (− 33.22), therefore Model B fit the data better.

**Table 2:** Comparison of SARIMA models

	Model A				Model B			
	β	SE(β)	T	*P*-value	β	SE(β)	T	*P*-value
AR1	-0.707	0.177	4.005	<0.001	0.945	0.027	35.645	<0.001
MA1	0.778	0.154	5.057	<0.001	-1.000	0.016	62.893	<0.001
SAR1	-0.454	0.060	7.576	<0.001	/			
SMA1	/				-0.688	0.057	12.148	<0.001
Log likelihood		19.61				34.98		
df		9				9		
*P*-value of Ljung-Box Q test		0.341				0.437		
AIC		-33.22				-63.96		

### SARIMAX model analysis and prediction

**Fig. 4:**
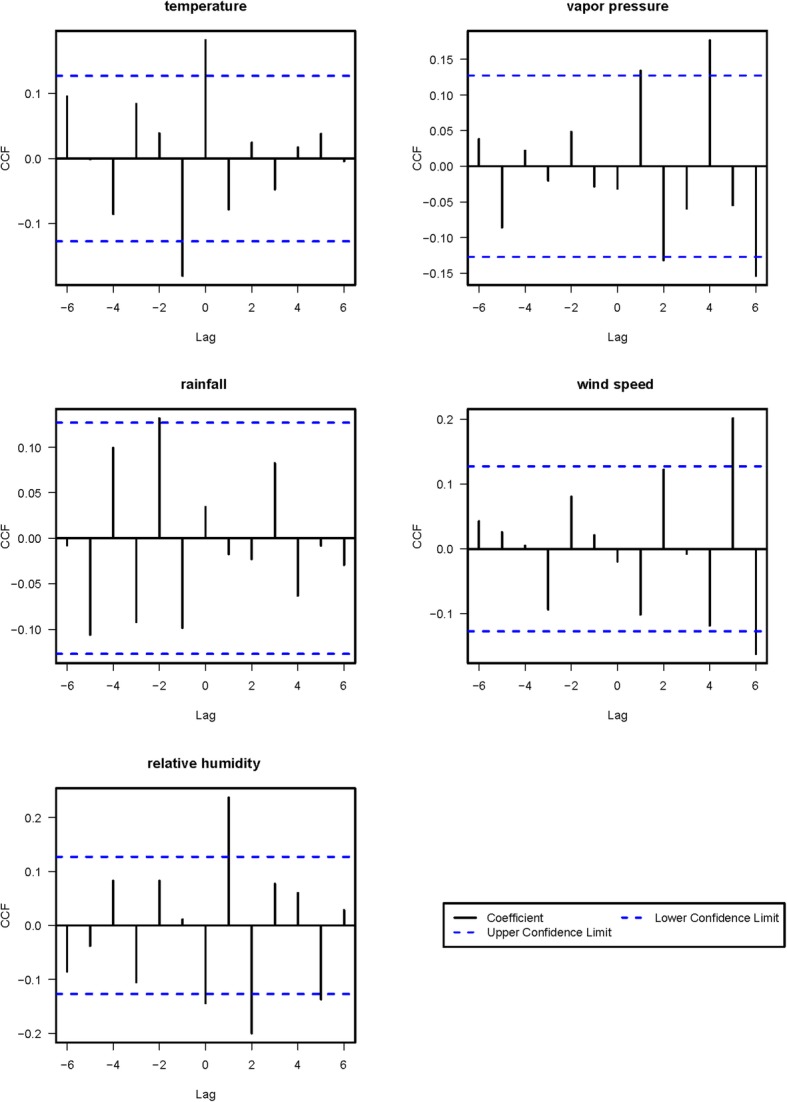
Correlations with pre-whitened weather variables

The CCF was used to investigate relationships between meteorological factors and mumps cases. The fitted SARIMA model was applied to pre-whiten the data for the monthly averages of the daily mean values of the meteorological factors. Figure 4 shows the cross-correlations between the pre-whitened meteorological variables (temperature, vapor pressure, rainfall, wind speed, and relative humidity) and logarithmically transformed numbers of mumps cases (log^mumps^) at lags of 1 to 6 months. All of the weather factors except rainfall were significantly associated with log^mumps^ at at least some of the lags. For example, the CCF for vapor pressure and log^mumps^ was significant at lags 1, 2, 4, and 6, and the CCF for wind speed and log^mumps^ was significant at lags 5 and 6. Those significant weather factors were added into the SARIMA model as covariates to establish the SARIMAX model. As shown in Table 3, two SARIMAX models with covariates yielded significant parameters and lowered the AIC value. This result indicated that mean temperature at lag 0 and vapor pressure at lag 2 affected log^mumps^ after fitting the time series regression model. Mean temperature at lag 0 (β = 0.016, *p* < 0.05, 95% confidence interval 0.001 to 0.032) was positively associated with log^mumps^, while vapor pressure at lag 2 (β = − 0.018, *p* < 0.05, 95% confidence interval ˗0.038 to ˗0.002) was negatively associated. SARIMAX (1, 1, 1) (0, 1, 1)_12_ with temperature at lag 0 was the optimal model with the lowest AIC value (Table 4).

**Table 3:** Comparison of SARIMA models with covariate

Model	Meteorological factors						AIC
	Variables	Lag	β	SE(β)	T	*P*-value	
SARIMA(1,1,1)(0,1,1)12	T	0	0.016	0.007	2.377	0.018*	-67.58**
SARIMA(1,1,1)(0,1,1)12	W	5	0.042	0.037	1.115	0.266	-63.20
SARIMA(1,1,1)(0,1,1)12	W	6	-0.073	0.037	1.952	0.052	-65.75**
SARIMA(1,1,1)(0,1,1)12	RH	0	-0.001	0.001	1.000	0.318	-62.95
SARIMA(1,1,1)(0,1,1)12	RH	1	0.002	0.001	1.769	0.078	-64.85**
SARIMA(1,1,1)(0,1,1)12	RH	2	-0.002	0.001	1.769	0.078	-64.92**
SARIMA(1,1,1)(0,1,1)12	RH	5	-0.001	0.001	0.429	0.669	-62.16
SARIMA(1,1,1)(0,1,1)12	V	1	0.005	0.009	0.517	0.606	-62.22
SARIMA(1,1,1)(0,1,1)12	V	2	-0.018	0.009	2.023	0.044*	-66.01**
SARIMA(1,1,1)(0,1,1)12	V	4	0.016	0.009	1.830	0.069	-65.20**
SARIMA(1,1,1)(0,1,1)12	V	6	-0.006	0.009	0.689	0.492	-62.43
SARIMA(1,1,1)(0,1,1)12	T	0	0.015	0.007	2.206	0.028*	-65.56**
	V	2	-0.016	0.009	1.862	0.064	

*T*: temperature; *W*: wind speed; *RH*: relative humidity; *V*: vapor pressure; *: *P* value < 0.05; **: AIC value < -63.96

**Table 4:** Description of SARIMAX model with mean temperature at lag 0

	β	SE(β)	T	*P*-value
AR1	0.945	0.026	35.932	<0.001
MA1	-1.000	0.016	62.893	<0.001
SMA1	-0.686	0.056	12.301	<0.001
Lag0 Temperature	0.016	0.007	2.377	0.018
Log likelihood	37.79			
df	9			
P-value of Ljung-Box Q test	0.545			
R-squared	0.617			
adjusted R-squared	0.612			
AIC	-67.58			

**Fig. 5:**
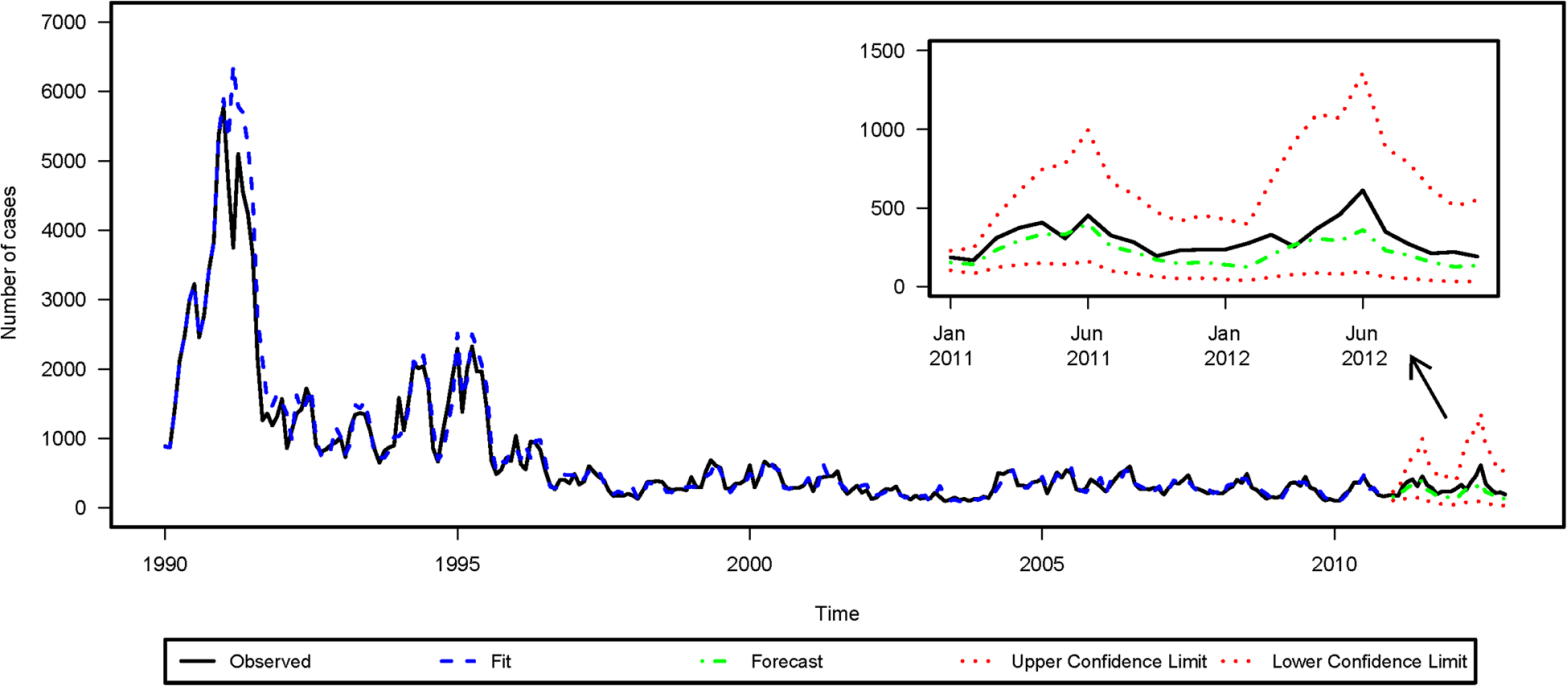
Prediction via the seasonal autoregressive integrated moving average model with explanatory variable

The SARIMAX model described above was used to attempt to retrospectively predict mumps cases from January 2011 to December 2012. The estimated and predicted results are shown in Fig. 5. The predicted monthly numbers of mumps cases all fell within the confidence intervals.

## Discussion

The incidences of many infectious diseases exhibit seasonal variation. In the present study the incidence of mumps cases in Beijing over a time-course exhibited clear seasonal effects. Cases peaked from late spring to early summer (April–July), and in winter (December–January). This is consistent with a study conducted by Li et al. [18] in Jining, China, in which large peaks were found in May and June, and in winter. In a study of the epidemiology of mumps conducted in China by Cui et al. [25] most mumps cases occurred between April and July, with a small peak occurring in November and December. Although the mechanisms underlying the seasonality of mumps incidence remain poorly understood, oscillatory changes in infectiousness, contact patterns, pathogen survival, host susceptibility, and population behaviors may contribute to the phenomenon [26, 27]. Seasonal variations in meteorological factors probably also play a role.

The results of the present study are similar to those of several previous studies investigating the effects of weather variables on mumps in Asia [15–18]. In all of those studies the occurrence of mumps was significantly associated with mean monthly temperature. In several studies there was an approximately linear association between mean temperature and mumps cases, over a certain temperature threshold. For example in Jining, a city in northern China, each 1 °C increase in mean temperature above 4 °C was associated with a 2.72% increase in the risk of mumps [18]. In Taiwan the number of mumps cases started to increase at a temperature of 20 °C, but began to decline when the temperatures exceeded approximately 25 °C [16]. Mumps virus is stable for days at 4 °C [1]. Higher temperatures are more conducive to the survival of mumps virus in the environment, and person-to-person contact [18]. Furthermore, in a study investigating correlations between meteorological conditions and physical activities performed in open-air settings, the number of individuals walking on a public track increased with temperature [28], which may also be indicative of increased outdoor activities more broadly. Partaking in frequent outdoor activities may increase the risk of mumps infection. In another study conducted in the United States, spring-break college travel was associated with an increase in mumps cases after 01 April [29].

In the present study vapor pressure at lag 2 was negatively associated with log^mumps^. In a study conducted in Taiwan the number of mumps cases began to increase at vapor pressures of 5–9 hPa and decreased at vapor pressures > 25–29 hPa [16]. The mechanism by which vapor pressure affects the transmission of mumps virus is poorly understood. Additional studies investigating the underlying mechanisms are warranted.

The survival of viruses outside the host depends partially on relative humidity. Viruses with lipid envelopes survive longer at lower relative humidity (20–30%) [30]. In addition, at higher wind speeds the spread of disease via respiratory droplets is rendered more likely [18]. In the present study, log^mumps^ was significantly correlated with relative humidity and with wind speed at different lags. The varying lag effects associated with weather parameters reported in other studies probably resulted from differences in study locations.

In the present study mean monthly temperature, relative humidity, vapor pressure, and wind speed at different lags were significantly associated with log^mumps^, with lag effects varying from 0 months to 6 months. After fitting the SARIMAX model, mean temperature at lag 0 was positively associated with log^mumps^, while vapor pressure at lag 2 was negatively associated. SARIMAX (1, 1, 1) (0, 1, 1)_12_ with temperature at lag 0 was the optimal model with the highest prediction accuracy, which overcame the hypothesis that the traditional time series model was linearly dependent on the variables included, and improved the accuracy of resulting predictions. The model was established based on mumps incidences and meteorological data in Beijing, China. Accordingly, it is only suitable for use to predict overall trends in Beijing.

The current study had some limitations. More meteorological parameters such as monthly mean, maximum, and minimum temperatures, sunshine duration, and other variables should be included in future studies to comprehensively evaluate relationships between meteorological parameters and mumps. Another limitation was that the mumps incidence data were only available on a per month basis. Weekly or daily incidence data may decrease the accuracy of lagged time estimation. Lastly, potentially confounding variables such as vaccine usage and school and household size may have affected mumps incidence. These data were not available for assessment in the current study.

## Conclusions

Various meteorological variables influence the incidence of mumps in Beijing, China. A time series regression model suggested that mean temperature at lag 0 and vapor pressure at lag 2 may influence log^mumps^. The utilization of a SARIMAX (1, 1, 1) (0, 1, 1)_12_ model with temperature at lag 0 is recommended for predicting the incidence of mumps in Beijing.

## Additional files

Additional file 1: **Table S1.** Monthly mumps data from January 1990 to December 2012, Beijing, China. (XLSX 15 kb)

Additional file 2: **Table S2.** Monthly means derived from daily average values of meteorological parameters measured from 1989 to 2012 in Beijing, China. (XLSX 26 kb)

## Abbreviations

ACF: autocorrelation function
AIC: Akaike information criterion
CCF: cross-correlation function
log^mumps^: logarithmically transformed numbers of mumps cases
PACF: partial autocorrelation function
SARIMA: seasonal autoregressive integrated moving average
SARIMAX: seasonal autoregressive integrated moving average model with explanatory variables

## References

[1] Hviid A, Rubin S, Mühlemann K (2008). Mumps. Lancet.

[2] MacDonald N, Hatchette T, Elkout L, Sarwal S (2011). Mumps is back: why is mumps eradication not working?. Adv Exp Med Biol.

[3] Vaccine preventable diseases monitoring system. http://www.who.int/immunization_monitoring/en/globalsummary/scheduleselect.cfm.]

[4] Zheng J, Zhou Y, Wang H, Liang X (2010). The role of the China experts advisory committee on immunization program. Vaccine.

[5] Majumder MS, Nguyen CM, Cohn EL, Hswen Y, Mekaru SR, Brownstein JS (2017). Vaccine compliance and the 2016 Arkansas mumps outbreak. Lancet Infect Dis.

[6] Trotz-Williams LA, Mercer NJ, Paphitis K, Walters JM, Wallace D, Kristjanson E, Gubbay J, Mazzulli T (2017). Challenges in interpretation of diagnostic test results in a mumps outbreak in a highly vaccinated population. Clinical and vaccine immunology: CVI.

[7] Pazdiora P, Skalova J, Kubatova A, Jezova I, Moravkova I, Podlesna I, Pruchova J, Spacilova M, Svecova M: [Mumps outbreak in the Plzen Region in 2011]. Epidemiologie, mikrobiologie, imunologie : casopis Spolecnosti pro epidemiologii a mikrobiologii Ceske lekarske spolecnosti JE Purkyne 2015, 64(4):242–249. 26795229

[8] Braeye T, Linina I, De Roy R, Hutse V, Wauters M, Cox P, Mak R (2014). Mumps increase in Flanders, Belgium, 2012-2013: results from temporary mandatory notification and a cohort study among university students. Vaccine.

[9] Cordeiro E, Ferreira M, Rodrigues F, Palminha P, Vinagre E, Pimentel JP (2015). Mumps outbreak among highly vaccinated teenagers and children in the central region of Portugal, 2012-2013. Acta Medica Port.

[10] Nedeljkovic J, Kovacevic-Jovanovic V, Milosevic V, Seguljev Z, Petrovic V, Muller CP, Hubschen JM (2015). A mumps outbreak in Vojvodina, Serbia, in 2012 underlines the need for additional vaccination opportunities for young adults. PLoS One.

[11] Whelan J, van Binnendijk R, Greenland K, Fanoy E, Khargi M, Yap K, Boot H, Veltman N, Swaan C, van der Bij A (2010). Ongoing mumps outbreak in a student population with high vaccination coverage, Netherlands, 2010. Euro surveillance: bulletin Europeen sur les maladies transmissibles = European communicable disease bulletin.

[12] Cui A, Zhu Z, Hu Y, Deng X, Sun Z, Zhang Y, Mao N, Xu S, Fang X, Gao H (2017). Mumps epidemiology and mumps virus genotypes circulating in mainland China during 2013-2015. PLoS One.

[13] Shuman EK (2011). Global climate change and infectious diseases. The international journal of occupational and environmental medicine.

[14] Falagas ME, Bliziotis IA, Kosmidis J, Daikos GK (2010). Unusual climatic conditions and infectious diseases: observations made by Hippocrates. Enferm Infecc Microbiol Clin.

[15] Yang Q, Yang Z, Ding H, Zhang X, Dong Z, Hu W, Liu X, Wang M, Hu G, Fu C (2014). The relationship between meteorological factors and mumps incidence in Guangzhou, China, 2005-2012. Human vaccines & immunotherapeutics.

[16] Ho YC, Su BH, Su HJ, Chang HL, Lin CY, Chen H, Chen KT (2015). The association between the incidence of mumps and meteorological parameters in Taiwan. Human vaccines & immunotherapeutics.

[17] Onozuka D, Hashizume M (2011). Effect of weather variability on the incidence of mumps in children: a time-series analysis. Epidemiol Infect.

[18] Li R, Lin H, Liang Y, Zhang T, Luo C, Jiang Z, Xu Q, Xue F, Liu Y, Li X (2016). The short-term association between meteorological factors and mumps in Jining, China. Sci Total Environ.

[19] Hubálek Z (2005). North Atlantic weather oscillation and human infectious diseases in the Czech Republic, 1951–2003. Eur J Epidemiol.

[20] Zhang DS, Zhang X, Ouyang YH, Zhang L, Ma SL, He J. Incidence of allergic rhinitis and meteorological variables: non-linear correlation and non-linear regression analysis based on Yunqi theory of chinese medicine. Chinese journal of integrative medicine. 2016. 10.1007/s11655-016-2588-9 27329149

[21] D.Cryer J, Chan K-S: Time Series Analysis with Applications in R, Second edn: Springer; 2008.

[22] Duan Y, Huang XL, Wang YJ, Zhang JQ, Zhang Q, Dang YW, Wang J (2016). Impact of meteorological changes on the incidence of scarlet fever in Hefei City, China. Int J Biometeorol.

[23] Zhang Y, Bi P, Hiller JE: Weather and the transmission of bacillary dysentery in Jinan, northern China: a time-series analysis. Public Health Rep (Washington, DC : 1974) 2008, 123(1):61–66. 10.1177/003335490812300109 PMC2099327 18348481

[24] Lin H, Yang L, Liu Q, Wang T, Hossain SR, Ho SC, Tian L (2012). Time series analysis of Japanese encephalitis and weather in Linyi City, China. International journal of public health.

[25] Cui A, Zhu Z, Chen M, Zheng H, Liu L, Wang Y, Ma Y, Wang C, Fang X, Li P (2014). Epidemiologic and genetic characteristics of mumps viruses isolated in China from 1995 to 2010. Infection, genetics and evolution: journal of molecular epidemiology and evolutionary genetics in infectious diseases.

[26] Fisman D (2012). Seasonality of viral infections: mechanisms and unknowns. Clinical microbiology and infection: the official publication of the European Society of Clinical Microbiology and Infectious Diseases.

[27] Fisman DN (2007). Seasonality of infectious diseases. Annu Rev Public Health.

[28] Suminski RR, Poston WC, Market P, Hyder M, Sara PA (2008). Meteorological conditions are associated with physical activities performed in open-air settings. Int J Biometeorol.

[29] Polgreen PM, Bohnett LC, Yang M, Pentella MA, Cavanaugh JE (2010). A spatial analysis of the spread of mumps: the importance of college students and their spring-break-associated travel. Epidemiol Infect.

[30] Tang JW (2009). The effect of environmental parameters on the survival of airborne infectious agents. J R Soc Interface.

